# Beloved Whiskers: Management Type, Care Practices and Connections to Welfare in Domestic Cats

**DOI:** 10.3390/ani10122308

**Published:** 2020-12-05

**Authors:** Daiana de Souza Machado, Luana da Silva Gonçalves, Rogério Ribeiro Vicentini, Maria Camila Ceballos, Aline Cristina Sant’Anna

**Affiliations:** 1 Núcleo de Estudos em Etologia e Bem-estar Animal, Departamento de Zoologia, Universidade Federal de Juiz de Fora, Juiz de Fora 36036-900, Brazil; daiana.machado@icb.ufjf.br (D.d.S.M.); luana_silva1500@hotmail.com (L.d.S.G.); rog.vicentini@hotmail.com (R.R.V.); 2 Department of Production Animal Health, Faculty of Veterinary Medicine, University of Calgary, Calgary, AB T2N 1N4, Canada; mariacamila.ceballos@ucalgary.ca

**Keywords:** behavioral problems, cat owners, feline welfare, indoor, outdoor

## Abstract

**Simple Summary:**

Little is known about the differences between indoor and outdoor cat management practices. Thus, our study investigated whether Brazilian cat owners’ management types were related to other cat care practices, the quality of human-animal interactions and cat welfare. We used social networks to distribute an online survey to cat owners. This survey included questions regarding owners’ sociodemographic data, type of management applied, cat care practices, and cat health and behavioral problems, as possible consequences of the management type. A total of 16,302 cat owners responded. Most (74.78%) owners reported providing indoor management for their cats; this corresponded to owners who lived in apartments and provided more cat care practices and interactions with their pets. Outdoor management was related to cats residing in farms or houses, sleeping outdoors, and having less interaction with their owners. We concluded that owners practicing indoor management were more likely to be closer to their cats than those reporting outdoor management, suggesting that the former may have more advantages related to closer human-animal relationships. It was noted, however, that indoor management was associated with obesity and owner-reported behavioral problems.

**Abstract:**

The quality of cat care practices depends in part on the type of management applied, which either positively or negatively impacts cat welfare. This study investigated whether the type of cat management (indoor vs. outdoor) was related to other cat care practices adopted by cat owners, associated with the quality of human-cat relationships and cat welfare. An online survey was distributed via social networks. Descriptive statistics, categorical Principal Component Analysis, Fisher’s Exact test and Chi-square test in contingency table were applied. A total of 16,302 cat owners returned the survey. Most Brazilian owners reported indoor management of their cats; this was related to owners living in apartments, more frequent use of cat care practices, and more interactions with their pets. Outdoor management was related to cats living in houses or farms, sleeping outdoors or around the neighborhood, and owners had fewer interaction with their pets. In conclusion, owners practicing indoor management seemed to be closer to their cats than owners reporting outdoor management. However, obesity and owner-reported behavioral problems were associated with indoor management.

## 1. Introduction

According to the Brazilian Institute of Geography and Statistics (Instituto Brasileiro de Geografia e Estatística, IBGE), the number of domestic cats in Brazilian households has recently increased [1]. Numbers of cats were 22 and 23.9 million in 2013 and 2018, respectively, an 8.1% increase [2]. Thus, in Brazil, cats were the companion animal with the largest increase in population. Although the number of cats as pets has increased, research assessing issues related to their management and care practices in the domestic context has been scarce in Brazil. Even globally, most cat-related studies were conducted in shelters, laboratories, and situations in which cats might impact the environment, such as natural areas, where cats’ predatory habits are a leading threat to the fauna [3].

Regarding the welfare of domestic cats, an issue that frequently leads to controversies and debates is how cats are maintained, i.e., indoor or outdoor management [4,5]. In the United States, it is recommended that cats are kept indoors, being wholly confined within a residence, without access to the street [6,7]. In contrast, in various countries, as Denmark, Chile, and the United Kingdom, most owners allow their cats to roam freely outside the house [5,6,8,9]. Finally, in Australia, there is a duality in the types of cat management, with half of the respondents of a survey conducted in 2016 (51%) reporting indoor management, and the other half reporting some level of outdoor management [10]. In a recent online survey in Brazil, more cat owners reported indoor than outdoor management [11].

It is expected that both management styles impose some risks and benefits [4]. For instance, risks related to outdoor cats include propagation of infectious diseases [11,12,13], unwanted pregnancies [4], motor vehicle accidents [14,15], fights with conspecifics [4], and mistreatment by people who do not like cats [16]. Outdoor cats might also impact the welfare of other animals, as wildlife due to predation [17,18], and niche overlap with wild carnivores [19]. Maintaining cats exclusively indoors has been associated with behavioral problems [8,20,21], obesity [22], fights (in multi-cat houses) [4], and accidents, including burns and intoxication with cleaning products [23]. Regarding the benefits, outdoor management might promote more mental stimulation, reduce boredom and frustration of a predictable environment, and stimulate more physical activities [23,24]. Indoor cats are regarded as being more protected against several risks that can be lethal, such as mistreatment, poisoning, and motor vehicle accidents [11,23].

Irrespective of the management type adopted by domestic cat owners, it is necessary to guarantee elevated levels of welfare through good cat care practices. Some authors suggest that an adequate human-cat relationship, perceived by the owners as positive, is beneficial for humans, leading them to promote a good quality of life for their cats [25,26,27]. In contrast, an inadequate relationship with a poor owner-cat bond might indicate few benefits to humans, and in some circumstances, cat care can be neglected [25]. However, there is a lack of knowledge about cat care practices and styles of cat management adopted by cat owners. For instance, do indoor owners subject their cats to certain specific care practices? Do those practices differ from those applied to outdoor cats? Answering these questions would provide insights regarding the impact of various styles of cat management on their welfare.

The aims of this study were to: (a) investigate whether the type of management (indoor vs. outdoor) is related to other cat care practices adopted by cat owners; and (b) understand if the type of management is associated with the quality of human-cat relationship and cats’ welfare. We hypothesized that: (i) owners reporting indoor management have closer contact with their cats and perform more cat care practices with a higher frequency of human-animal interactions; and (ii) cats kept indoors are at greater risk of some welfare issues related to poor body condition (obesity) and behavioral problems compared to outdoor cats.

## 2. Materials and Methods

### 2.1. Ethical Statement

An online questionnaire was sent to cat owners. The study involved non-identified respondents; thus, we followed the Brazilian Ethical Standards of Scientific Research Involving Human Subjects (Resolution n° 510/2016 of the National Health Board). Ethical goals were attained by advising respondents of the aims of the study, so that they could make an informed decision about their participation. Anonymity and confidentiality were assured. Respondents were also informed that their participation did not imply any type of financial or other compensation and that they could withdraw from answering the questionnaire at any time.

### 2.2. Questionnaire Structure and Application

A 39-question questionnaire for Brazilian cat owners was developed in Portuguese, based on published papers describing feline management [6,7,8,9]. The questionnaire comprised multiple-choice and forced-choice questions, in addition to open-ended questions related to respondents’ information. Only part of the data was used in this paper. Three sets of closed questions were included: (i) Cat owners’ sociodemographic data; (ii) type of management (indoor vs. outdoor) and other cat care practices; and; (iii) cats’ physical and mental health aspects. The questionnaire is in Supplementary Materials File S1.

A convenience sampling was performed. Respondents were recruited by sending the questionnaire link via social networks (e-mail, Facebook™, Instagram™, and WhatsApp™). The free online survey tool ‘Google Forms’ (Google™) was used. Respondents were allowed to participate only if they met the criterion of owning at least one cat. Data collection occurred from 10 June to 11 July 2020. Prior to analysis, the dataset was cleaned, removing answers considered dubious based on the participant’s age (must be >18 years) and the number of cats (zero cats-not having cats at the moment) (*n* = 389). Respondents that owned several cats were required to answer based on the one owned for the longest amount of time.

### 2.3. Statistical Analyses

The questionnaire contained continuous (cat age), ordinal, and nominal data (multiple choice questions). Thus, categorical principal component analyses (PCA) were used. Similar to a conventional PCA, this reduces the data dimensionality to fewer new variables, i.e., the principal components (or dimensions) [28,29,30]. Categorical PCA is similar to the PCA for continuous data regarding aims, results, and interpretations. However, categorical PCA is more appropriate for distinct types of variable scales, i.e., qualitative (nominal and ordinal) variables and quantitative variables [28,29,30]. Two PCA were performed, one including the type of management and cat care practices, and the other including the type of management and health and behavioral problems. Nominal variables in the first PCA were: type of management, reason for outdoor management, type of residence, use of litter box, and cat obtention. In addition, ordinal variables were: where cat stays when owner leaves, where cat stays when owner is at home, where cat sleeps, frequency of owner leaving, frequency that owner plays with the cat, buys gifts or toys for the cat, brushes the cat and cuts the cat’s claws. The second PCA included the following nominal variables: cat sex, type of management, use of therapeutic diet, cat neutering, health problems, and behavioral problems; whereas cat body condition score, frequencies of visits to a vet, and frequency of cat vaccination were included as ordinal variables, in addition to cat age as a continuous scale. In both PCA, principal components with eigenvalues >1 were retained, and variables with loadings >0.30 were considered the highest contributions to the components.

Complementary to PCA, we also used a Chi-square test in a contingency table (or Fisher’s Exact test in 2 × 2 tables), to estimate associations between type of management (indoor or outdoor) with all other variables related to cat care practices, cat health, and behavioral problems. All statistical analyses were conducted with SPSS Statistics Software, Version 21.

## 3. Results

### 3.1. Sociodemographic Data of Owners and Their Cats

**Figure 1:**
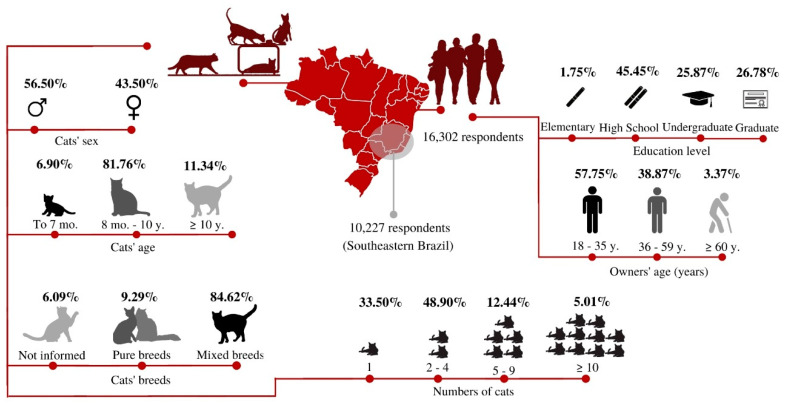
Sociodemographic data of owners and their cats.

Responses from 16,302 owners were obtained, most of them female respondents 93.22% (15,197), ranging from 18 to 35 years old, with a high school education (Figure 1). There were responses from 26 Brazilian States and the Federal District, with most of the answers from the southeast region of Brazil (62.73%), where almost half (42.2%) of the Brazilian population is located (Supplementary Materials Table S2). Most owners had more than one cat, with an average of 1.89, ranging from 1 to 130 cats. Most animals were mixed-breed, females, ranging from 8 months to 10 years old (Figure 1).

### 3.2. Association between the Management (Indoor vs. Outdoor) with Other Cat Care Practices

**Figure 2:**
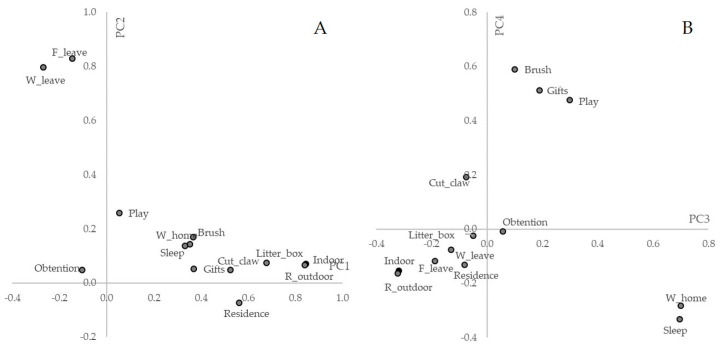
Plot of variables used in the principal component analysis for the type of management, cat care practices, and human-cat interactions. (**A**). Plot of principal components 1 and 2. (**B**). Plot of principal components 3 and 4. Acronyms refer to the following variables: ‘Frequency the owner leaves home’ (F_leave), ‘Where the cat stays when the owner leaves’ (W_leave), ‘Cat’s place when the owner is at home’ (W_home), ‘Cat obtention form’ (Obtention), ‘Frequency of playing with the cat’ (Play), ‘Place where the cat sleeps’ (Sleep), ‘Brushing frequency’ (Brush), ‘Claw cutting frequency’ (Cut_claw), ‘If owner buys gifts for the cat’ (Gifts), ‘Residence type’ (Residence), ‘Litter box availability’ (Litter_box), ‘Reasons for outdoor access’ (R_outdoor), and ‘Type of management’ (Indoor).

The relationships between management and care practices were initially analyzed using a multivariate exploratory technique, the categorical PCA. The first four principal components (PC) explained 54.58% of the total variance of the dataset (Supplementary Materials Table S3). PC1 explained 23.65% of the variance (eigenvalue 3.07) and had higher positive loadings for the type of management (indoor), reasons for outdoor management, availability of litter box, type of residence, cuts cats’ claws, buys gifts and toys, brushes the cat, where the cat stays when the owner is at home and where the cat sleeps (Figure 2A). PC2 explained 11.41% of the variance in the dataset (eigenvalue 1.48) and had higher positive loadings for where the cat stays when the owner leaves the house and frequency of the owner leaving the house.

PC3 explained 10.65% of the variance (eigenvalue 1.38), and the variables with higher positive loading were: where the cat stays when the owner is at home, where the cat sleeps, and plays with the cat. Variables with higher negative loadings were: type of management (indoor) and reason for outdoor management (Figure 2B). Finally, PC4 explained 8.87% of the variance (eigenvalue 1.15), and variables with higher positive loadings were: brushes the cat, buys gifts and toys, and plays with the cat. The variable where the cat slept had higher negative loading.

The most common type of management reported was indoor (74.78%, 12,191/16,302). According to the Chi-square test (or Fisher’s Exact), indoor management was significantly associated with a higher frequency of the following cat care practices: (a) residence in apartments; (b) cats adopted; (c) cats who sleep indoors, including in owner’s room/bed; (d) when the owner is at home, the cat stays in the same place with owners, as the sofa or bed; (e) owners who leave the house regularly and the cat stays alone; (f) the cat stays indoor with access to all of the house when the owner leaves; (g) owners frequently buy gifts for the cat; (h) play with the cat once a day; (i) frequently cuts cat’s claws; (j) brushes cat often; and (k) provides litter box, compared to cats kept outdoors (Table 1, comparison within each line).

Outdoor management was reported by 25.22% (4111/16,302) of owners. From owners reporting outdoor access, most (72.31%, 3176/4392) answered that the layout of their house did not enable them to limit the cats’ movements, whereas 27.69% (1216/4392) considered this necessary and beneficial for the cat. There was a significant association between outdoor management and a higher frequency of the following answers: (a) households in houses or farms; (b) cats that appeared at owners’ houses (i.e., stray cats that were taken in) or were given to them; (c) cats that slept outdoors or around the neighborhood; (d) the owner leaves, but the cat does not stay alone; (e) when the owner leaves, the cat stays outdoors or around the neighborhood; (f) occasionally buys gifts or cat toys; (g) play with the cat never or occasionally; (h) play with the cat several times a day; (i) does not cut cats claws, because it is not necessary; (j) does not brush the cat; and (k) does not provide litter box or provides it but the cat does not use, compared to indoor cats’ owners (Table 1, comparison within each line).

### 3.3. Association between the Type of Management (Indoor vs. Outdoor) with Cat Welfare

**Figure 3:**
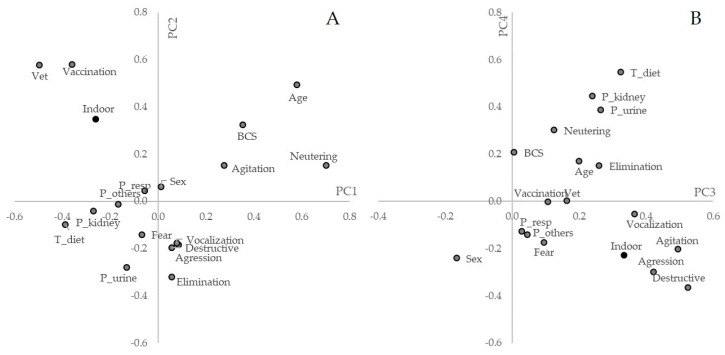
Plot of variables used in the Principal Component Analysis for the type of management, cats’ health, and behavioral problems. (**A**). Plot of principal components 1 and 2. (**B**). Plot of principal components 3 and 4. Acronyms refer to the following variables: ‘Vet visits’ (Vet), ‘Vaccination frequency (Vaccination), ‘Type of management’ (Indoor), ‘Respiratory problems’ (P_resp), Other health problems (P_others), ‘Kidney problems’ (P_kidney), ‘Urinary problems’ (P_urine), ‘Use of therapeutic feed’ (T_Diet), ‘Cat sex’ (Sex), ‘Cat body composition score’ (BCS), ‘Neutering’ (Neutering), ‘Cat’s age (Age), ‘Excessive vocalization’ (Vocalization), ‘Excessive agitation’ (Agitation), ‘Destructiveness behavior’ (Destructive), ‘Aggressiveness (Agression), ‘Excessive fear’ (Fear) and ‘Inadequate elimination of feces and urine’ (Elimination).

The relationships between management with health and behavioral problems were first analyzed using a categorical PCA. The first five PC explained 39.13% of the total variance in the dataset (Supplementary Materials Table S3). PC1 explained 9.84% of the variance (eigenvalue 1.77) and had higher positive loadings for cat neutering, cat age, and body condition score (BCS), and the higher negative loadings were frequency of visits to the vet, use of therapeutic diet, and frequency of vaccination (Figure 3A). PC2 explained 8.36% of the variance (eigenvalue 1.50), with higher positive loadings for the frequency of visits to the vet, frequency of vaccination, cat age, type of management (indoor), BCS, and the higher negative loading was inadequate elimination.

PC3 explained 7.65% of the variance (eigenvalue 1.38), with higher positive loadings for destructive behavior, agitation, aggression, vocalization, type of management indoors, and use of therapeutic diet (Figure 3B). PC4, explaining 7.08% of the variance (eigenvalue 1.27), had the following variables with higher positive loadings: use of therapeutic diet, kidney diseases, urinary problems, and neutering; variables with higher negative loadings were destructive behavior and aggression. Finally, PC5 explaining 6.20% of the variance (eigenvalue 1.12), had higher loadings for cat sex, other health problems, kidney problems, fear, and BCS; and respiratory and urinary problems with higher negative loadings.

According to the Chi-square (or Fisher’s) tests in contingency tables, indoor management was significantly associated with a higher frequency of destructive behavior, agitation, excessive fear, overweight and obesity, neutering, kidney problems, respiratory problems, other health problems, use of therapeutic diet, vaccination of the cat and visits to the vet, compared to outdoor cats (Table 2, comparison within each line). In contrast, outdoor management was related to a higher frequency of inappropriate elimination of urine and feces and standard body condition.

## 4. Discussion

This study provided a survey regarding cat care practices and their relationship with cats’ welfare, reported by a large owner sample. The majority (~75%) of owners reported maintaining their cats indoors. Among owners reporting outdoor management, most of them declared that their house did not provide physical or structural conditions to limit cats’ movements. A lower percentage of owners reporting outdoor management justified that they allowed the cat to exit the house because it could be a healthy habit and positive for the cat, consistent with a previous study from our research group [7]. It was possible to classify the types of management and cat-owner interactions in two distinct and extreme styles. At one extreme, owners who maintained their cats indoors had a closer relationship with their cats, reported a higher frequency of cat care practices, such as brushing and cutting claws, plus more frequent clinical preventive care, characterizing a closer management style. At the other extreme, for owners who reported outdoor handling, their cats did not stay in the same places as the owners, the cats were kept outdoors when the owner left the house, and they reported a lower frequency of cat care practices, characterizing an extensive management style. We inferred that the style of management was characterized by a set of traits, including more than simply allowance of outdoor access.

In relation to the type of management and cat care practices, the first PCA revealed that the most important variables to characterize the variability in the management practices were: type of handling, followed by reasons for allowing outdoor access, type of residence, place where the cat slept, provision of litter box, the owner buys gifts and toys for the cat, brushes the cat and cuts claws. Indoor management was related to the variables higher frequency of residence in apartments, cats who sleep indoors including the owner’s room/bed, owners provide litter boxes, buys gifts for the cat frequently, brushes the cat, and cuts cat’s claws. Some previous studies identified residence as a factor that influenced the type of management [9,11]. Indoor management was related to areas with intense urbanization and apartments [9,11]. Conversely, outdoor cats were more frequent in areas with lower population densities, houses with yards, suburban and rural areas, all conditions in which it can be difficult to restrict cats’ movements [9,11], consistent with the present study. It is important to highlight that the associations described in this study do not characterize cause and effect relationships. For instance, people that live in apartments are more likely to be in urban areas, which are unsuitable for an outdoor cat. Concerning the place within the house where the cat stays when the owner is at home, 93.6% of indoor cats were reported to stay in the same places as the owner (including sofas and beds), evincing a close human-animal contact. It is possible that in apartments, likely smaller places, cats spent more time with the owner, explaining in part these associations. For cats with few enrichment resources in small households, the owner becomes a valuable resource, considered a social-environmental enrichment, as suggested by previous studies [31,32]. For outdoor cats, the percentage was also high (85%) but lower than indoor animals. For outdoor cats, the frequencies of animals who stayed outdoors or indoors but not in the same places as humans were higher than indoor cats. In fact, some studies suggested that how cats establish bonds and interact with their owners might differ as a function of the space and resources available [32,33], which should influence owners’ allowance and cats’ choices of where to stay when owners are present.

In the second PC, variables with higher loadings were the place where the cat stayed when the owner left and the frequency with which the owner left the house, characterizing a component related to owner absence. Owners’ frequency and duration of leaving the house can be characterized as a possible risk factor for separation-related problems [20]. For owners reporting indoor management, 44.2% of the participants left the house daily and their cats stayed alone. Perhaps for people living in apartments, there was a higher frequency of single occupants, which means the cat was left on its own more frequently. Most (85.8%) reported that the cats were left indoors with access to the entire house. Of the owners reporting outdoor management, only 28.6% left the cat alone daily, but 53.4% allowed the cats to have access to the entire house, whereas 27.4% left the cat outdoors when the owner was absent. These cats exposed to outdoor access, without the presence of the owner, could be more exposed to risks such as fights or accidents.

In PC3, variables with higher loadings were the place where the cat stayed when the owner was present, the place where the cat slept, the owner playing with the cat, type of management, and the reasons for allowing outdoor access. Owners reporting indoor management had a higher frequency of cats staying in the same place as the owner, playing with the cat once a day, and allowing cats to sleep in the owners’ beds, characterizing a closer human-cat style of management. The owners reporting outdoor management had a higher frequency of cats staying outdoors when the owner was at home, cats sleeping outdoors, never or occasionally playing with the cat, or playing several times a day, compared to indoor cats, suggesting another extreme of the more extensive style of management. Despite the significant difference in the Chi-square test, the association between type of management and play with the cat was not straightforward, since owners of outdoor cats had higher frequencies for the lowest (never play) and the highest frequency of play (several times a week). Owners sharing their own bed or bedroom with their pets has become a common human behavior in modern society in various places of the world, revealing a closer relationship with these animals [34,35]. The choices of where to stay and sleep can also be related to cat’s behaviors and preferences, not exclusively an owner’s management decision. Our results corroborated previous findings reporting a higher frequency of human-animal interactions and closer contact for cats kept indoors [7,33]. Both of these aspects were related to a closer human-cat management style.

PC4 had higher loadings for some variables that had already contributed to other principal components, reinforcing their importance to characterize the management style. Variables such as where the cat slept, plays with the cat, buys gifts and toys for the cat, and brushes the cat had higher loadings in this PC. Aspects related to human-cat interactions have an important role in the welfare of both, and the quality and quantity of positive interactions result in cat welfare improvements in any context (i.e., household or shelters) [36]. Domestic cats can benefit from positive interactions with humans [31,37], and they can even perceive humans as part of their social environment [37,38,39]. It can be expected that positive interactions with owners (such as playing behavior) improve the welfare of both indoor and outdoor cats. In the first case, the lack of mental stimulation (typical of a captive environment) could promote boredom and frustration, requiring additional efforts to enrich the environment. However, positive contacts with a familiar human can be considered environmental enrichment for indoor cats. For outdoor cats, a previous study [7] reported that owners of free-roaming cats had lower frequency of positive interactions and close contacts with their cats and even neglected the necessity of interacting positively with them. According to Grigg and Kogan [7], an owner-cat bond score was significantly higher for indoor cats than for cats allowed to roam freely, which was also associated with a higher frequency of behavioral problems for outdoor cats. However, a causal relationship between outdoor management and behavioral problems was not established [7]. This can explain, in part, our results related to higher frequency of buying gifts and toys for the cat, cat care practices (brushes cat and cuts claws) and closer physical proximity (sharing the same places, like the sofa or bed) for cats defined in this study as having a closer management style, compared to the extensive management style. Even though brushing and cutting claws is aversive for some cats, both are recommended cat care practices, with welfare implications [40,41]. Brushing helps in removing dead hair and, consequently, avoids the formation of hairballs [41]. Although intolerable to some cats, cutting cats’ claws reduces the risk of injuries from aversive interactions between cats in multi-cat houses. Claw trimming can be accepted by cats if done gently and from kittenhood [40,42]. Thus, all these interactions with outdoor cats could reinforce their bonds with humans.

A second PCA was applied for type of management in relation to physical and mental health (behavioral problems), generating five principal components. Although the percentage of variance explained by this analysis was not high (39.13%), relationships between variables on the PCs were interesting. In PC1, cat castration, age, BCS, use of therapeutic diet, frequency of visits to the vet, and vaccination were variables with higher loadings, characterizing a component related to cat health. The type of management had higher loadings in PC2, related to the frequency of visits to the vet, frequency of vaccinations, age, BCS, and inappropriate elimination. Owners reporting indoor management had higher frequencies of obesity and overweight scores in BCS, more visits to vet and higher frequency of cat vaccination, which could be related to more clinical preventative care in the indoor cats. Higher vet visit frequency for indoor cats may be associated with problems due to cat obesity and overweight. In PC3, there were higher loadings for behavioral problems such as aggressiveness, excessive vocalization, destructiveness, and agitation, in addition to the type of management and use of therapeutic diet, characterizing a relationship between the type of management and behaviors. Indoor cats had higher frequencies of destructiveness, agitation, and the use of therapeutic diet, compared to outdoor cats, but they did not differ for aggressiveness or excessive vocalization. The PC4 had some variables related to behaviors (destructiveness and aggression) and others related to physical health (use of therapeutic diet, kidney diseases, urinary problems, and neutering). However, it did not have any relationship with management. Finally, PC5 had higher loadings for cat sex, other health problems, kidney problems, fear, BCS, respiratory and urinary problems, but did not reveal any relation with the type of management.

The BCS had higher loadings in the first two PCs, representing an important characteristic related to the type of management and cat welfare. Cats kept indoors were more likely to be defined by their owners as overweight or obese. Conversely, owners reporting outdoor management were more likely to characterize their cats with the standard score. This result corroborated previous studies indicating that indoor cats were frequently associated with obesity, given the lack of stimulation and environmental predictability, which leads to a lower level of physical activity [22,23,43]. Another possible explanation could be that owners of indoor cats provided more treats as gifts, since they reported buying things for the cat more frequently, including sachets and snacks, in addition to toys, beds, or other gifts.

Clinical preventative care practices, such as frequent visits to the vet and frequent vaccinations, were also more frequent for cats maintained indoors. Neutering can also be considered a preventative practice, more frequently reported by owners of indoor than outdoor cats. Neutering was also related to cats’ age, therapeutic diet, and other clinical preventative care practices in PC1 of the PCA, often conducted in domestic cats [44]. In a previous study conducted with Brazilian cat owners (*n* = 8485) in 2019, 65.7% of the owners declared having neutered their cats [11]. In the present study, the frequency of neutered cats was even higher (87.7%). The high frequency of neutering in the present study was probably due to the dissemination of its preventative benefits, such as avoiding unwanted pregnancies, reduction in cats’ home ranges, reduction in reproductive tract diseases, and lower frequency of behavioral problems, e.g., urine marking [4,45,46]. It was reinforced by the association between neutering and other clinical preventative care practices (frequent visits to the vet and vaccinations). Owners declaring they do not neuter their cats also had lower chances of vaccinating and deworming their cats regularly, as reported in previous studies [11,45]. Possibly, it reflected a lack of concern about the possible consequences of not neutering or performing clinical preventative care, or even due to financial and/or cultural reasons [45].

Inappropriate elimination had higher negative loading in PC2, demonstrating a higher frequency for outdoor than indoor cats. This result was similar to a previous study reporting a higher frequency of inappropriate elimination for outdoor cats [45]. These findings should be related to urine marking, given that cats kept outdoors are more likely to have encounters with other conspecifics, and males tend to mark their territory vertically [47]. The frequency of neutering was lower in outdoor cats, what could also lead to more urine marking. Another plausible explanation was that house soiling could be considered unacceptable for owners, characterizing a possible motivation to maintain the cat outside the house. Based on the available data, it is impossible to know if inappropriate elimination was a consequence of or led to outdoor management. In PC2, cat age also had a higher loading, with a higher frequency of kittens and also of old cats for indoor than for outdoor cats. Perhaps the lethal risks related to outdoor management, such as car accidents [14,15], poisoning and mistreatment [4,16,24] or infectious diseases [4,11,24,48] could be related to the lower occurrence of cats >10 years old in outdoor management.

The PC3 had higher loadings for therapeutic diet and behavioral problems (aggressiveness, excessive vocalization, destructiveness, and agitation), most of them with higher frequency for indoor than outdoor cats. A possible explanation might be that owners could be more observant of cats’ behaviors due to the proximity with them in indoor conditions. Previous studies have related indoor management with a higher risk of behavioral problems such as aggressiveness, agitation, excessive vocalization, inadequate elimination, apathy, destructiveness, and excessive fear [8,20,49,50,51]. In general, captive environments tend to lack stimulation, being monotonous and predictable. Otherwise, indoor housing might include aversive stimuli for the cats, for example, lack of hiding places and exposure to unwanted interactions with unfamiliar humans, children, or other animals, without the possibility to escape when confined [23,49]. In those cases, owners must recognize cats’ necessities and individuality to provide positive stimulation, protection from unwanted stimuli, environmental enrichment to prevent behavioral problems, and consequently improve cat welfare [52,53]. Despite lacking scientific support, routine outside walks using a leash have been recommended to provide positive mental stimulation and physical activity for some individuals habituated to this practice [40]. However, benefits of walking on a leash would depend on the cat’s temperament, as this could be perceived as frightening for fearful and timid cats. Studies should be conducted to determine if there are benefits of walking on a leash for indoor cats to prevent behavioral problems and improve welfare.

The behavioral problems more frequently reported in cats kept indoors (destructiveness, and agitation) are usually characterized as indicative of behavioral disorders related to general anxiety [54,55]. Anxiety is characterized by intense and unpleasant sensations and feelings of anticipation of a danger stimulus not present [55,56]. When these behaviors occur during the owner’s absence, they could indicate separation-related problems [20,57]. It is interesting to note that owners of indoor cats reported a higher frequency of them leaving the house on a daily basis and the cat staying alone during their absence, compared to outdoor cats. Thus, the possible association of indoor management with the likelihood of factors predisposing cats to separation-related problems must be further investigated [20,57]. For instance, the frequency of owners’ absences and lack of environmental enrichment were factors previously related to the occurrence of separation-related problems in cats [20]. It is important to consider behavioral problems as the main cause of cats’ abandonment and relinquishment in several countries, contributing to the high numbers of animals in shelters [58,59,60,61]. There are cases in which these animals can be euthanized if not adopted, reinforcing the necessity of investigations related to the risk factors of behavioral problems in owned cats [60,61,62].

The use of a therapeutic diet was related to most of the principal components of the PCA (except by PC2). This variable was related to health and behavioral problems and had higher frequency for indoor than outdoor cats. The use of therapeutic diet was related to kidney and urinary problems. Perhaps, for cats kept indoors, the owner would have more opportunities to observe cat urine and eliminatory behavior and perceive this kind of problem better than for cats kept outdoors, leading to this relationship. Additionally, therapeutic diet is also recommended for animals who are overweight and obese for weight control [63]; thus, indoor management could be more indirectly related to the therapeutic diet, given the higher occurrence of cats with excessive BCS.

This study had some limitations that must be acknowledged. Firstly, we could not collect data from owners without access to the internet, leading to bias in our sample. In Southeast Brazil, 84.8% of the houses have access to internet, whereas in the Northeast, only 69.1% have access, which might explain different sampling of these regions (Supplementary Materials Table S2). Our sample had an educational condition better than the average Brazilian population (Supplementary Materials Table S2) which could be related to a better socioeconomic condition than the Brazilian population in general. It may also have affected our frequencies of responses about the type of management and cat care practices that can be dependent on several owner-related traits, including educational level and financial condition. For example, we speculate that an owner with a better economic situation would spend more money on preventative medical care for their cats. A second limitation was related to the subjectivity of the owner when answering some of the questions, mainly those related to health and behavioral problems. Some behaviors are normal for domestic cats (e.g., scratching), but could be perceived as a ‘problem’ by owners, leading them to report ‘destructiveness’. Conversely, conditions less readily perceived, such as urinary and kidney problems, could be underestimated in the questionnaire. Another point is that the questionnaire did not include questions about the relationship between cats in multi-cat households and about the provision of environmental enrichment; these would have helped with interpretation of some results about the relationships between indoor management and behavioral problems. Regarding outdoor cats, it would be useful to include questions regarding the neighborhood’s security, for example, proximity to streets or roads with heavy motor vehicle traffic. Despite all those limitations, the online questionnaire method enabled us to gather a large sample of owners, with a wide geographic coverage (all Brazilian states) and which, during a short interval, would not be feasible using other methodologies of data collection.

In summary, pet owners usually have strong emotional connections to their animals, even considering them part of their families [7,11,64,65]. Thus, they tend to provide protection, care, positive interactions, and comfort, leading to positive impacts on welfare [64]. However, not all owners are the same in terms of cat care practices and styles of management. They might vary depending on many cat-owner related aspects. Regardless of the style of management adopted by a cat owner, good cat care practices must be adopted. In this study, we identified traits that could be used to define styles of cat management ranging in a continuum, in which the two extremes were defined as close management and extensive management. Closer management did not necessarily mean ‘positive management,’ but the owners had more intense contact with their animals. Both management styles might lead to benefits and risks for cat welfare, impacting cats’ quality of life. For example, closer management could compromise welfare as a function of how the cat perceives the close contact and interactions with humans, in addition to excessive predictability and lack of stimulation typical of a confined environment. Furthermore, it might lead to fear and anxiety, behavioral problems, and conflicts with conspecifics in multi-cat indoor environments [20,51,66,67,68]. Other risks for cats kept exclusively indoors are obesity and domestic accidents [22,23,43]. In turn, whereas an extensive management style should present some benefits by stimulating natural behaviors, it also might impose risks if the care practices and necessity of protection are not considered. Cats that stay outdoors without owner oversight are more exposed to several potentially lethal conditions, e.g., motor vehicle accidents, mistreatment, fights with conspecifics, and dog attacks [11,12,14,16,43,69]. The close versus extensive management are examples of extremes and many owners may be in an intermediate part of this continuum.

There is little published information about cat management by Brazilian owners. This initial research is important to identify cat care practices in the sampled population and is useful in supporting responsible ownership education. Future research is recommended to further investigate the welfare implications of various management styles. In this study, management types were limited to indoor vs. outdoor, but alternative types between these two extremes should be considered, for example, cats allowed to roam free during the day but confined at night. It is also recommended that future studies collect a representative sample of the Brazilian population by including people without internet access.

## 5. Conclusions

Three quarters of the Brazilian cat owners sampled reported indoor cat management. Among those reporting outdoor management, the primary reason was the layout of their house, not allowing them to limit their cats’ movements. We identified two different extreme styles of cat care practices and human-cat interactions. One extreme was characterized as a closer management style, reflected by owners who maintained their cats exclusively indoors. Those were more likely to report residing in apartments, the cat sleeping in the owner’s room/bed, the cat staying in the same places as the owners when owners are at home (as sofa and beds), owners who leave the house regularly while their cat stays alone with access to all of the house, owners buy gifts and frequently brush the cat, cut claws more frequently and provide a litter box, compared to cats kept outdoors. The other extreme, characterized as extensive management style, reflected owners who maintained their cats outdoors, households in houses or farms, with the cat sleeping outdoors or around the neighborhood, when the owner leaves the house the cat does not stay alone, or the cat stays outdoors when the owner leaves, and owners only occasionally buying gifts or toys. These owners did not cut claws, nor brush, and did not provide litter boxes. Clinical preventative care practices (frequent visits to the vet, frequent vaccinations, and neutering) were more frequent for indoor cats. Finally, cats maintained indoors had higher frequencies of owner-reported behavioral problems and obesity, whereas outdoor cats had a higher frequency of inappropriate elimination. Thus, both management styles offer risks and benefits to cats’ welfare and the human-cat relationship. It is necessary, regardless of the type of management adopted, to meet cats’ requirements and good care practices to avoid welfare problems associated with each management style.

## Figures and Tables

**Table 1:** Absolute and relative frequencies (%, within parentheses) of cat care practices and human-cat interactions for cat population sampled (total) and for cats kept indoors and outdoors. Chi-square tests (or Fisher’s Exact tests in 2 × 2 table) are shown to test the association between each practice and the type of management (indoor vs. outdoor). Significant values are shown in bold.

Cat Care Practices	Total (*n* = 16,302)	Indoor (*n* = 12,191)	Outdoor (*n* = 4111)	χ^2^	*p*-Value
**Type of residence**					
Farm	338 (2.1)	149 (1.2)	189 (4.6)	**2111.847**	**<0.001**
House	10,413 (63.9)	6704 (55.0)	3709 (90.2)		
Apartment	5551 (34.1)	5338 (43.8)	213 (5.2)		
**Reasons for outdoor access**					
In my opinion, it is necessary	1216 (7.5)	179 (1.5)	1037 (25.2)	**14795.152**	**<0.001**
The layout of my house does not allow me to limit the cat’s movements	3176 (19.5)	119 (1.0)	3057 (74.4)		
My cat is kept indoors	11,910 (73.1)	11,893 (97.6)	17 (0.4)		
**Acquisition mode**					
I adopted him/her	6142 (37.7)	5082 (41.2)	1114 (27.1)	**741.326**	**<0.001**
He/she appeared at my house	1780 (10.9)	946 (7.8)	834 (20.3)		
I bought him/her	349 (2.1)	326 (2.7)	23 (0.6)		
He/she was given to me	2283 (14.0)	1535 (12.6)	748 (18.2)		
I adopted as a stray cat	5748 (35.3)	4356 (35.7)	1392 (33.9)		
**Where does the cat sleep**					
Outdoors or around the neighborhood	804 (4.9)	420 (3.4)	384 (9.3)	**252.791**	**<0.001**
Indoors except in my room	1468 (9.0)	1036 (8.5)	432 (10.5)		
Indoors, including in my room/bed	14,030 (86.1)	10,735 (88.1)	3295 (80.2)		
**Where does the cat stay when you are at home**					
Outdoors	363 (2.2)	146 (1.2)	217 (5.3)	**359.467**	**<0.001**
Indoors, hidden under beds and wardrobes	334 (2.0)	189 (1.6)	145 (3.5)		
Indoors, but not in the same places where I stay	705 (4.3)	449 (3.7)	256 (6.2)		
In the same places where I stay, e.g., beds and sofas	14,900 (91.4)	11,407 (93.6)	3493 (85.0)		
**Do you leave home daily**					
I leave and the cat stays alone	6566 (40.3)	5390 (44.2)	1176 (28.6)	**353.758**	**<0.001**
I leave but he/she does not stay alone	6233 (38.2)	4220 (34.6)	2013 (49.0)		
I do not leave home daily	3503 (21.5)	2581 (21.2)	922 (22.4)		
**Where does the cat stay when you leave**					
Outdoors or around the neighborhood	1442 (8.8)	316 (2.6)	1126 (27.4)	**2869.042**	**<0.001**
Indoors and confined in any room	528 (3.2)	458 (3.8)	70 (1.7)		
Indoors with access to entire house	12,651 (77.6)	10,456 (85.8)	2195 (53.4)		
I almost never go out	1681 (10.3)	961 (7.9)	720 (17.5)		
**Buy gifts/cat toys**					
Yes, frequently	12,449 (76.4)	9819 (80.5)	2630 (64.0)	**501.186**	**<0.001**
Occasionally	3504 (21.5)	2202 (18.1)	1302 (31.7)		
No, never	349 (21.1)	170 (1.4)	179 (4.4)		
**Play with the cat**					
Never/occasionally	987 (6.1)	668 (5.5)	319 (7.8)	**63.299**	**<0.001**
Two or three times a week	1423 (8.7)	1074 (8.8)	349 (8.5)		
Once a day	3531 (21.7)	2788 (22.9)	743 (18.1)		
Several times a day	10,361 (63.6)	7661 (62.8)	2700 (65.7)		
**Do you cut the cat’s claws**					
No, because it is not necessary	5984 (36.7)	3596 (29.5)	2388 (58.1)	**1550.931**	**<0.001**
No, he/she does not allow	3782 (23.2)	2690 (22.1)	1092 (26.6)		
Yes, frequently	6536 (40.1)	5905 (48.4)	631 (15.3)		
**Do you brush the cat**					
No, I do not brush my cat	3770 (23.1)	2336 (19.2)	1434 (34.9)	**545.484**	**<0.001**
Occasionally	6132 (37.6)	4558 (37.4)	1574 (38.3)		
Yes, often	6400 (39.3)	5297 (43.5)	1103 (26.8)		
**Do you provide a litter box**					
No	2137 (13.1)	563 (4.6)	1574 (38.3)	**3772.111**	**<0.001**
Yes, but he/she does not use	744 (4.6)	303 (2.5)	441 (10.7)		
Yes, he/she has and uses it	13,421 (82.3)	11,325 (92.9)	2096 (51.0)		

**Table 2:** Absolute and relative frequencies (%, within parentheses) of health aspects and behavioral problems for the cat population sampled (total) and cats kept indoors and outdoors. Chi-square tests (or Fisher’s exact tests in 2 × 2 table) are shown to test the association between each problem and management type (indoor vs. outdoor). Significant values are shown in bold.

Cats Demographic Data, Health, and Behavioral Problems	Total (*n* = 16,302)	Indoor (*n* = 12,191)	Outdoor (*n* = 4111)	χ^2^	*p*-Value
**Age**					
Kitten (≤7 mo)	1125 (6.9)	943 (7.7)	182 (4.4)	**80.264**	**<0.001**
Adult	13,329 (81.1)	9786 (80.3)	3543 (86.2)		
Senior (>10 y)	1848 (11.3)	1462 (12.0)	386 (9.4)		
**Gender**					
Female	9212 (56.5)	6940 (56.9)	2272 (55.3)	-	0.064
Male	7090 (43.5)	5251 (43.1)	1839 (44.7)		
**Destructive behavior**					
Yes	2551 (15.6)	2098 (17.2)	453 (11.0)	**-**	**<0.001**
No	13,751 (84.4)	10,093 (82.8)	3658 (89.0)		
**Excessive vocalization**					
Yes	799 (4.9)	610 (5.0)	189 (4.6)	-	0.316
No	15,503 (95.1)	11,581 (95.0)	3922 (95.4)		
**Inappropriate elimination of urine and feces**					
Yes	1316 (8.1)	929 (7.6)	387 (9.4)	**-**	**<0.001**
No	14,986 (91.9)	11,262 (92.4)	3724 (90.6)		
**Aggressiveness**					
Yes	1444 (8.9)	1098 (9.0)	346 (8.4)	-	0.254
No	14,858 (91.1)	11,093 (91,0)	3765 (91.6)		
**Agitation**					
Yes	2104 (12.9)	1697 (13.9)	407 (9.9)	**-**	**<0.001**
No	14,198 (87.1)	10,494 (86.1)	3704 (90.1)		
**Excessive fear**					
Yes	1960 (12.0)	1540 (12.6)	420 (10.2)	**-**	**<0.001**
No	14,342 (88.0)	10,651 (87.4)	3691 (89.8)		
**Body condition score (BCS)**					
Thin	1201 (7.4)	907 (7.4)	294 (7.2)	**54.443**	**<0.001**
Standard	9087 (55.7)	6639 (54.5)	2448 (59.5)		
Overweight	5205 (31.9)	3968 (32.5)	1237 (30.1)		
Obese	809 (5.0)	677 (5.6)	132 (3.2)		
**Neutered**					
Yes	14,291 (87.7)	10,887 (89.3)	3404 (82.8)	**-**	**<0.001**
No	2011 (12.3)	1304 (10.7)	707 (17.2)		
**Kidney problems**					
Yes	440 (2.7)	374 (3.1)	66 (1.6)	**-**	**<0.001**
No	15,862 (97.3)	11,817 (96.9)	4045 (98.4)		
**Urinary problems**					
Yes	607 (3.7)	440 (3.6)	167 (4.1)	-	0.182
No	15,695 (96.3)	11,751 (96.4)	3944 (95.9)		
**Respiratory problems**					
Yes	382 (2.3)	319 (2.6)	63 (1.5)	**-**	**<0.001**
No	15,920 (97.7)	11,872 (97.4)	4048 (98.5)		
**Other health problems**					
Yes	1729 (10.6)	1409 (11.6)	320 (7.8)	**-**	**<0.001**
No	14,573 (89.4)	10,782 (88.4)	3791 (92.2)		
**Therapeutic diet**					
Yes	2102 (12.9)	1633 (13.4)	469 (11.4)	**-**	**<0.001**
No	14,200 (87.1)	10,558 (86.6)	3642 (88.6)		
**Vaccination and deworming of the cat**					
Always	13,281 (81.5)	10,219 (83.6)	3602 (74.5)	**196.063**	**<0.001**
Occasionally	2700 (16.6)	1794 (14.7)	906 (22.0)		
Never	321 (2.0)	178 (1.5)	143 (3.5)		
**Visits to vet**					
Always	5309 (32.6)	4530 (37.2)	779 (18.9)	**696.516**	**<0.001**
Occasionally	8137 (49.9)	5975 (49.0)	2162 (52.6)		
Never	2856 (17.5)	1686 (13.8)	1170 (28.5)		

## Supplementary Materials

The following are available online at https://www.mdpi.com/2076-2615/10/12/2308/s1: File S1: Questionnaire for cat owners (Supplementary Material S1). Table S2: Sociodemographic data of the Brazilian population according to the IBGE and in the sampled population (Supplementary Material S2). Table S3: Loadings of variables obtained in the two principal component analyses (PCA) (Supplementary Material S3).
