# Supplementary material for: Beloved Whiskers: Management Type, Care Practices and Connections to Welfare in Domestic Cats
Source: Animals (Basel). 2020 Dec 5;10(12):2308. doi: 10.3390/ani10122308 (PMC7762120; doi:10.3390/ani10122308)
Supplement: Supplementary file 1 [file animals-10-02308-s001.zip › Questionnaire for cat owners.docx]

**Questionnaire for cat owners**

Owner’s sociodemographic data

1. What is your first name? ___________________________________­­­­­­­­­­­­­­______________
2. Which Brazilian State do you live? _________________________________________
3. Which city do you live? __________________________________________________
4. How old are you?

( ) ≤ 18.

( ) 18 to 35.

( ) 36 to 59.

( ) ≥ 60.

1. Gender?

( ) Female. ( ) Male.

1. Educational level:

( ) Ellementary school.

( ) High school.

( ) Undergraduate.

( ) Graduate (Lato sensu).

( ) Graduate (Stricto senso).

1. Which type of residence do you live in?

( ) House. ( ) Apartment. ( ) Farm. ( ) Others.

1. How many cats do you have? ____________________________________________

**Please, answer based on the cat owned for the longest interval:**

1. Cat Sex:

( ) Male. ( ) Female.

1. How old is your cat?

( ) Kitten (by 7 months).

( ) Adult (between 8 months to 10 years).

( ) Old (older than 10 years).

About your relationship with your cat, answer:

1. How did you obtain your cat?

( ) He/she appeared at my house.

( ) I adopted as a stray cat.

( ) I adopted him/her.

( ) He/she was given to me.

( ) I bought him/her.

1. Does your cat leave the house, i.e., he/she walks on roofs, neighbors' yards or in the street?

( ) Yes, he/she has outdoor access.

( ) No, he/she only goes for a walk with a leash and guide, under supervision.

( ) No, he/she does not have outdoor access, he/she is kept indoor, and I do not take him for a walk.

1. If you answered yes to the previous question, answer why does he have outdoor access?

( ) Because I allow and consider necessary and healthy to walk around the neighborhood.

( ) The layout of my house does not allow me to limit the cat’s movements.

1. Is your cat neutered?

( ) Yes. ( ) No.

1. Which is your cat's breed? __________________________________________
2. Does your cat have any of the problems below?

( ) Urinary problems.

( ) Kidney problems.

( ) Diabetes.

( ) Allergies.

( ) Respiratory problems.

( ) Tumors.

( ) Gastrointestinal problems.

( ) Others.

( ) None.

1. According to this image, which option best characterizes your cat's body condition?


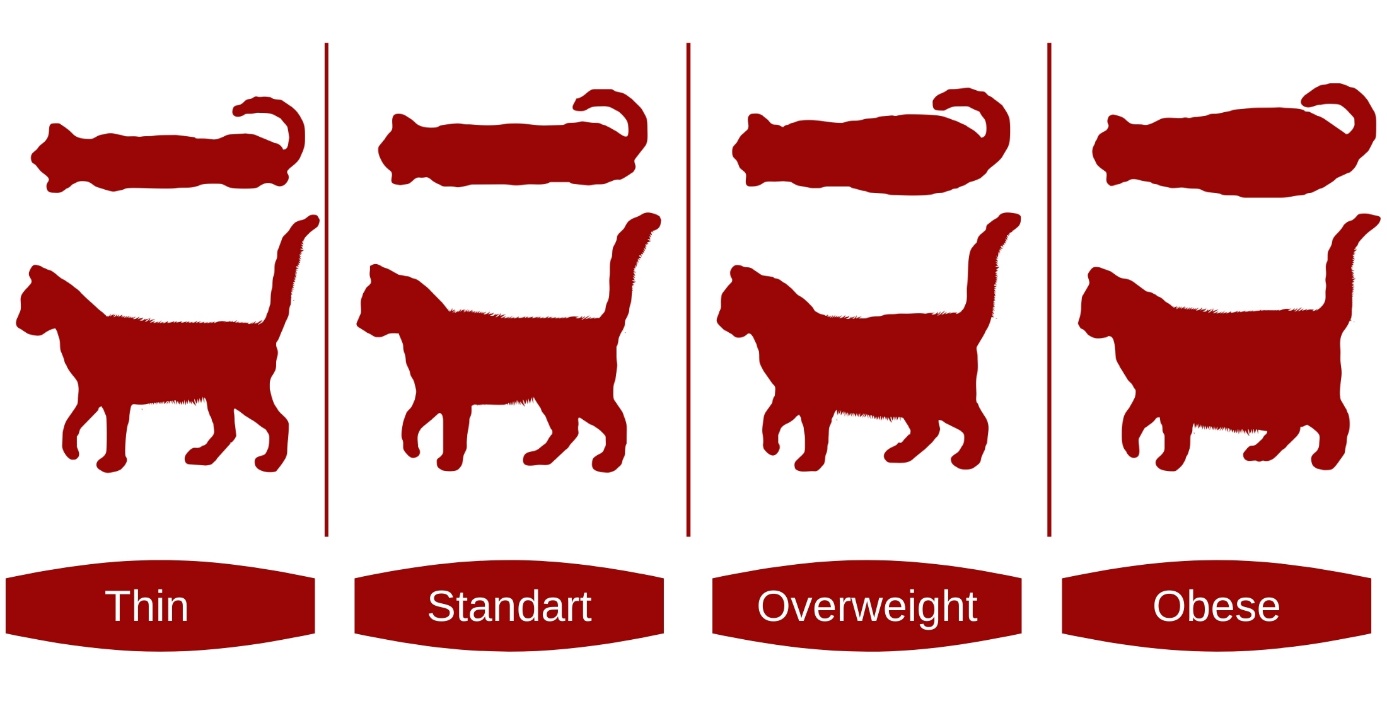


( ) Thin. ( ) Standard. ( ) Overweight. ( ) Obese.

1. Does your cat eat any therapeutic diet?

( ) Yes. ( ) No.

1. Where does your cat sleep?

( ) Indoors except in my room / bedroom.

( ) Indoors, including in my room/bed or anywhere else he/she chooses.

( ) Outdoors or around the neighborhood.

1. Do you buy things for your cat, such as sachets, snacks, toys, beds or other gifts?

( ) Yes, frequently. ( ) Occasionally. ( ) No, never.

1. Does your cat perform any of the following behaviors?

( ) Excessive meowing (meows excessively, even disturbing the residents of the house or neighbors).

( ) Destructive scratching behavior (he scratches furniture, curtains, even destroying them).

( ) Aggressiveness (tries to attack or bite or scratch other cats, dogs or people).

( ) Elimination of urine and feces in inappropriate places (urine or defecate on the bed, sofas, owner's clothes, owner's shoes, pillows).

( ) Agitation (moves a lot, runs around the house, is restless).

( ) Excessive fear (he is very shy, runs away whenever visitors arrive at the house, spends a lot of time hiding under the bed, wardrobes or other hiding places).

( ) None.

1. How often do you play with your cat?

( ) Several times a day.

( ) Once a day.

( ) Two or three times a week.

( ) Occasionally.

( ) I don't play with my cat.

1. Do you take your cat to the vet frequently?

( ) Always, to prevent diseases.

( ) Occasionally.

( ) I never take him to the vet.

1. Do you provide vaccines, dewormers, anti-flea and anti-ticks for your cat?

( ) Always.

( ) Occasionally.

( ) Never.

1. Do you brush your cat?

( ) Yes, at least once a day.

( ) Yes, at least once a week.

( ) Occasionally.

( ) No, I do not brush my cat.

1. Do you cut your cat's claws?

( ) Yes, frequently.

( ) No, he/she does not allow.

( ) No, because it is not necessary.

1. Do you provide a litter box?

( ) Yes, he/she has and uses the litter box.

( ) Yes, he/she has a litter box but does not use it.

( ) He/she does not have, he/she does his needs inside the house.

( ) He/she does not have, he/she makes his needs outdoors, in the garden or in the yard.

1. When you are at home, where does your cat stay most of the time?

( ) Indoor, in the same places where I stay, e.g. beds and sofas.

( ) Indoors, but not in the same places where I stay.

( ) Indoors, hidden under beds and wardrobes.

( ) Outdoors.

1. Do you leave the home daily to work / study and leave your cat alone?

( ) Yes, I leave and the cat stays alone.

( ) Yes, I leave but he/she does not stay alone, there are other persons in the house.

( ) I do not leave home daily.

1. When do you leave home to work / study where your cat stay?

( ) Indoors with access to entire house.

( ) Indoors and confined in any room.

( ) He/she stays outdoors until I return (e.g.: on the terrace, on the balcony, garden or yard).

( ) He/she walks around the neighborhood during my absence.

( ) I almost never go out.
