# Supplementary material for: Beloved Whiskers: Management Type, Care Practices and Connections to Welfare in Domestic Cats
Source: Animals (Basel). 2020 Dec 5;10(12):2308. doi: 10.3390/ani10122308 (PMC7762120; doi:10.3390/ani10122308)
Supplement: Supplementary file 1 [file animals-10-02308-s001.zip › Supplementary Material 2-resubmitted.docx]

| **Characteristic** | **Participants N (%)** | **IBGE Census Data (%)** | |
| --- | --- | --- | --- |
|  |  | PNADC (2019) | |
| **Gender** |  |  | |
| Female | 15,197 (93.22) | 51.8 | |
| Male | 1,063 (6.52) | 48.2 | |
|  |  |  |  |
| **Age** |  |  | |
| ≥ 17 | - | 24,2 | |
| 18-59 | 1,5751 (96.62) | 60.1 | |
| ≥ 60 | 551 (3.38) | 15.7 | |
|  |  |  |  |
| **Education level** |  |  | |
| Illiterate | - | 6.4 | |
| Elementary incomplete | - | 32.2 | |
| Elementary | 285 (1.75) | 12.5 | |
| High School | 4,218 (25.87) | 31.4 | |
| Undergraduate | 7,409 (45.45) | 17.4 | |
| Graduate | 4,366 (26.78) |  |  |
|  |  |  |  |
| **Region of residence within Brazil** |  |  | |
| Southeast | 10,227 (62.73) | 42.2 | |
| Center-West | 771 (4.73) | 7.7 | |
| South | 3,747 (22.99) | 14.3 | |
| North | 463 (2.84) | 8.6 | |
| Northeast | 1,094 (6.71) | 27.2 | |
|  |  |  | |
|  |  | PNADC (2018) | |
| **Internet access in residence per region** |  |  | |
| Southeast | . | 84.8 | |
| Center-West | . | 83.5 | |
| South | . | 81.8 | |
| North | . | 72.1 | |
| Northeast | . | 69.1 | |
| IBGE: Brazilian Institute of Geography and Statistics; PNADC: Continuous National Household Sample Survey. Avalilable online: https://biblioteca.ibge.gov.br/visualizacao/livros/liv101736_informativo.pdf and https://biblioteca.ibge.gov.br/visualizacao/livros/liv101707_informativo.pdf | | | |

**Table S2**. Socio-demographic data of the Brazilian population according to the IBGE and in the sampled population.
