# Supplementary material for: Beloved Whiskers: Management Type, Care Practices and Connections to Welfare in Domestic Cats
Source: Animals (Basel). 2020 Dec 5;10(12):2308. doi: 10.3390/ani10122308 (PMC7762120; doi:10.3390/ani10122308)
Supplement: Supplementary file 1 [file animals-10-02308-s001.zip › Supplementary Material 3-resubmitted.docx]

**Table 1** - Loadings of variables used in the Principal Component Analysis for the type of management, cat care practices, and human-cat interactions. Variables in bold were considered the highest contributions to the components.

| **Variables** | **PC1** | **PC2** | **PC3** | **PC4** |
| --- | --- | --- | --- | --- |
| Type of management - Indoor | **0.84** | 0.07 | **-0.32** | -0.16 |
| Reasons for outdoor access | **0.84** | 0.07 | **-0.32** | -0.16 |
| Type of residence | **0.56** | -0.07 | -0.08 | -0.13 |
| Where does the cat stay when you leave | -0.27 | **0.80** | -0.13 | -0.08 |
| Where does the cat stay when you are at home | **0.35** | 0.14 | **0.70** | -0.28 |
| Where does the cat sleep | **0.33** | 0.14 | **0.70** | **-0.33** |
| Do you provide a litter box | **0.68** | 0.07 | -0.05 | -0.02 |
| Do you leave home daily | -0.15 | **0.83** | -0.19 | -0.12 |
| Acquisition mode | -0.10 | 0.05 | 0.06 | -0.01 |
| Play with the cat | 0.05 | 0.26 | **0.30** | **0.48** |
| Buy gifts/cat toys | **0.37** | 0.05 | 0.19 | **0.51** |
| Do you brush the cat | **0.37** | 0.17 | 0.10 | **0.59** |
| Do you cut the cat's claws | **0.52** | 0.05 | -0.08 | 0.19 |
| Eigenvalues | 3.07 | 1.48 | 1.38 | 1.15 |
| Variance (%) | 23.65 | 11.41 | 10.65 | 8.87 |

**Table 2** - Loadings of variables used in the Principal Component Analysis for the type of management, cats' health, and behavioral problems. Variables in bold were considered the highest contributions to the components.

| **Variables** | **PC1** | **PC2** | **PC3** | **PC4** | **PC5** |
| --- | --- | --- | --- | --- | --- |
| Type of management - Indoor | -0.26 | **0.35** | **0.34** | -0.23 | 0.03 |
| Gender | 0.01 | 0.06 | -0.17 | -0.24 | **0.45** |
| Age | **0.58** | **0.49** | 0.20 | 0.17 | -0.05 |
| Body condition score (BCS) | **0.35** | **0.32** | 0.01 | 0.21 | **0.35** |
| Visits to vet | **-0.50** | **0.58** | 0.16 | 0.00 | -0.06 |
| Vaccination and deworming of the cat | **-0.36** | **0.58** | 0.11 | 0.00 | -0.17 |
| Therapeutic diet | **-0.39** | -0.10 | **0.32** | **0.55** | 0.19 |
| Neutered status | **0.70** | 0.15 | 0.13 | **0.30** | 0.03 |
| Kidney problems | -0.27 | -0.04 | 0.24 | **0.45** | **0.38** |
| Urinary problems | -0.13 | -0.28 | 0.26 | **0.39** | **-0.35** |
| Respiratory problems | -0.06 | 0.04 | 0.03 | -0.13 | **-0.37** |
| Other health problems | -0.17 | -0.01 | 0.05 | -0.14 | **0.40** |
| Excessive fear | -0.07 | -0.14 | 0.09 | -0.17 | **0.36** |
| Aggressiveness | 0.09 | -0.18 | **0.42** | **-0.30** | 0.08 |
| Inappropriate elimination of urine and feces | 0.06 | **-0.32** | 0.26 | 0.15 | -0.11 |
| Agitation | 0.28 | 0.15 | **0.49** | -0.20 | -0.03 |
| Excessive vocalization | 0.08 | -0.18 | **0.37** | -0.05 | 0.05 |
| Destructive behavior | 0.06 | -0.20 | **0.53** | **-0.36** | -0.02 |
| Eigenvalues | 1.77 | 1.50 | 1.38 | 1.27 | 1.12 |
| Variance (%) | 9.84 | 8.36 | 7.65 | 7.08 | 6.20 |
